# SOX2/SOX17 Molecular Switching by Polyphenols to Promote Thyroid Differentiation in 2D and 3D Models of Anaplastic Thyroid Cancer †

**DOI:** 10.3390/biology14121730

**Published:** 2025-12-02

**Authors:** Fabiola Vaglica, Mattia Biondo, Giuseppe Siragusa, Giorgio Arnaldi, Valentina Guarnotta, Giuseppe Pizzolanti, Laura Tomasello

**Affiliations:** 1 Dipartimento di Promozione della Salute, Materno-Infantile, di Medicina Interna e Specialistica di Eccellenza “G. D’Alessandro” (Promise), University of Palermo, Piazza delle Cliniche, 2, 90127 Palermo, PA, Italy; fabiola.vaglica@unipa.it (F.V.); giorgio.arnaldi@unipa.it (G.A.); giuseppe.pizzolanti@unipa.it (G.P.); laura.tomasello@unipa.it (L.T.); 2 Dipartimento di Scienze e Tecnologie Biologiche Chimiche e Farmaceutiche (Stebicef), University of Palermo, Viale delle Scienze, Ed. 16, 90128 Palermo, PA, Italy; mattia.biondo@unipa.it (M.B.); giuseppe.siragusa01@unipa.it (G.S.); 3 Advanced Technologies Network (ATeN) Center, Viale delle Scienze-Edificio 18/A, 90128 Palermo, PA, Italy

**Keywords:** anaplastic thyroid cancer, resveratrol, SOX2/SOX17 balance, differentiation, 3D culture, nutraceuticals

## Abstract

Deep alterations in tumor cell gene profiles resulting in the loss of their specific functions are frequently the cause of resistance to traditional cancer treatments. Therefore, reprogramming the expression pattern of cancer cells toward a differentiated phenotype represents a promising therapeutic strategy. In this study, we investigated whether resveratrol (RSV) and its natural analogs—3,4′,5-trimethoxystilbene (3-MET-OX) and isorhapontigenin (ISOR-H-PG)—can modulate the SOX2/SOX17 balance and promote re-differentiation in anaplastic thyroid cancer (ATC) cells. Two human ATC cell lines (SW1736 and 8505c) and non-tumoral thyroid cells (Nthy-ori 3-1) were cultured in two-dimensional (2D) or three-dimensional (3D) systems and treated with polyphenols at sub-cytotoxic doses. In 2D cultures, cell viability and cell cycle analyses confirmed a cytostatic effect characterized by G1 arrest. In 3D cultures, polyphenol treatment caused morphological disruption of ATC spheroids and significantly modulated the gene expression profile. RSV and 3-MET-OX reduced stemness markers (SOX2, NANOG), increased the thyroid lineage transcription factor (SOX17), and enhanced differentiation genes (TTF-1, TPO, NIS). Overall, these results support our hypothesis that modulation of the SOX2/SOX17 ratio by polyphenols provides a mechanistic basis for re-differentiation, thereby improving therapeutic responsiveness in ATC.

## Simple Summary

One of the most aggressive types of thyroid cancer, anaplastic thyroid cancer, frequently resists standard therapies like chemotherapy, radiotherapy, and surgery. This resistance mainly results from the loss of the normal gene expression profile of thyroid cells, which acquire features like cancer stem cells (CSCs). This behavior has been linked to an altered balance between two key regulatory genes, SOX2 and SOX17, which determine whether a cell remains undifferentiated or becomes specialized. In this study, we investigated whether natural plant-derived compounds known as polyphenols (resveratrol and two of its natural analogs) could help thyroid cancer cells regain normal functional characteristics and become more responsive to conventional therapies. Using a three-dimensional culture model that more accurately reproduces the architecture and growth conditions of human tumors, we found that these compounds slow cell proliferation and promote a transition from a stem-like to a more differentiated cell type by rebalancing SOX2 and SOX17 expression levels. These findings suggest that polyphenols may act as supportive agents for standard treatments and could open new perspectives for the management of highly aggressive thyroid cancers.

## 1. Introduction

Anaplastic thyroid cancer (ATC) is a rare and undifferentiated endocrine tumor without reliable therapies and poor prognosis. Due to severe early metastasis and a rapid fatal course, surgery is rarely performed, while radiotherapy and chemotherapy exhibit low efficacy. Although new approaches (e.g., immunotherapy) are now available for some patients, therapy resistance issues persist and are largely attributed to a loss of thyroid cell function due to the downregulation of thyroid terminal differentiation genes, among them sodium-iodide symporter (NIS), thyroid transcription factor-1 (TTF)-1, and thyroid peroxidase (TPO). Disrupting the circuit that sustains the acquisition of a de-differentiated state in ATC remains the main challenge to resensitize cells to conventional or novel therapies. One possible strategy involves targeting cancer stem cells (CSCs) [1,2,3,4,5].

CSCs are a subpopulation of tumor cells related to relapses and resistance to therapies, leading to cancer mortality, and characterized by high plasticity. We previously identified an ATC-derived CSCs subpopulation characterized by high expression of several stem cell markers, such as Sry-related HMG box (SOX) 2, Octamer-binding transcription factor (OCT) 4, and homeobox protein NANOG (NANOG). A potential upstream role of SOX2 in regulating cell proliferation and tumor progression was proposed, suggesting that this gene could overcome chemotherapy resistance [6,7]. In contrast, it is known that SRY-related HMG-box 17 (SOX17) exhibits anti-tumoral properties in papillary thyroid cancer (PTC); indeed, its low expression is positively correlated with increased PTC cell migration and invasion. Moreover, the activity of SOX17 has been studied in some tumor models and appears to function as a tumor suppressor. Although this evidence, the impact of the SOX family on thyroid cancer remains unclear [8,9]. It has been proven that specific nutraceuticals can modulate in ATC, the expression of some of the master genes involved in thyroid differentiation: for example, resveratrol (RSV) activates Notch1 and up-regulates paired box (PAX) 8, TTF-1 and NIS in HTh7 and 8505c cells, while the isoflavone genistein increases thyroglobulin (Tg), NIS, thyroid peroxidase (TPO), thyroid-stimulating hormone receptor (TSHR) and TTF-2 in SW1736 and 8505c; quercetin can also induce NIS and reduce the dedifferentiation marker CD97 in ATC models [10,11,12]. Our previous in vitro studies showed that RSV, a natural polyphenol, could affect the stem cell features, promoting the differentiation towards the epithelial lineage by modulation of the SOX2/SOX17 balance in limbal primary mesenchymal stem cells. In the present study, two ATC cell lines, SW1736 and 8505c, were used to investigate the effects of RSV and two of its natural analogs, 3,4′,5-trans-trimethoxystilbene (3-MET-OX) and isorhapontigenin (ISOR-H-PG) [13].

Our goal was to evaluate the regulation of SOX17 and SOX2 and the expression of specific thyroid markers (e.g., NIS, TPO, TTF-1), indicating a possible differentiation-inducing role of the nutraceutical treatments on ATC cell lines.

To better mimic an in vivo tumor microenvironment, 3D cell culture systems were employed along with the traditional monolayer cultures. In 3D cell culture systems, increased cell–cell and cell-extracellular matrix interactions, different access to oxygen, nutrients, and drugs, lead to a better simulation of the tumor microenvironment occurring in vivo [14]. Thus, to demonstrate and confirm a possible antitumor effect of polyphenols, spheroidal cell structures of thyroid cell lines were used to replicate a tumorigenesis process, assessing morphological or molecular changes after treatments.

Our work aims to provide further contributions to the understanding of the regulatory dynamics of de-differentiation in ATC, with a particular focus on the effect of natural compounds in reverting this process and promoting therapy re-sensitization for this aggressive cancer.

## 2. Materials and Methods

### 2.1. Cell Lines and Culture Conditions

Three commercial thyroid cell lines were selected for the experimentation: one thyroid follicular epithelial cell line, Nthy-ori 3-1, and two thyroid cancer cell lines, SW1736 and 8505c (American Type Culture Collection (ATCC), Manassas, VA, USA).

#### 2.1.1. Two-Dimensional (2D) Monolayer Cultures

Conventional 2D monolayer cells were cultured in Roswell Park Memorial Institute medium (RPMI) high glucose medium (EuroClone Spa, Milan, Italy) supplemented with 10% fetal bovine serum (FBS; EuroClone Spa, Milan, Italy) and 5% GlutaMax (Thermo Fisher Scientific, Monza, Italy). Cultures were maintained in a humidified incubator (5% CO_2_ and 37 °C).

#### 2.1.2. Three-Dimensional (3D) Spheroid Cultures

To assess 3D spheroid cultures, Nthy-ori 3-1, SW1736 and 8505c cells were cultured in complete RPMI supplemented with 0.1 mg/mL Geltrex™ (Thermo Fisher Scientific, Waltham, MA, USA). Cell suspensions (1.0 × 10^4^ cells/cm^2^) were seeded into ultra-low attachment 24-well plates (Corning Costar, Corning, NY, USA). Plates were incubated at 37 °C and 5% CO_2_, allowing spheroids to form spontaneously within 14 days. The medium was gently replaced every 2–3 days to maintain viability. Spheroid formation and growth progression were followed by optical microscopy observations (Leica DMI300B, Leica, Wetzlar, Germany). Images were acquired by Leica Suite application software (LAS Core version 4.4.0) and analyzed via NIS-Elements BR software (version 6.20.00, by Nikon, Tokyo, Japan) to obtain data related to spheroids radius: three images from three different experiments were analyzed for every 3D condition, and at least five spheroids were measured from every image. Results were expressed in μm (mean ± SD).

### 2.2. Reagents and Polyphenol Treatments

Resveratrol (RSV) was purchased from SIGMA (Prod No. R5010, CAS number: 501-36-0, Sigma-Aldrich, Merck KGaA, Darmstadt, Germany). One mg powder was dissolved in 0.438 mL of DMSO to prepare a 10 mM stock solution. 3,5,4′-trimethoxy-trans-stilbene (3-MET-OX) was purchased from MedChemExpress (Prod No. HY-N1408, CAS: 22255-22-7, Monmouth Junction, NJ, USA). One mg powder was dissolved in 0.369 mL of DMSO to prepare a 10 mM stock solution. Isorhapontigenin (ISOR-H-PG) was purchased from SIGMA (Prod No. SML0590, CAS number: 32507-66-7, Sigma-Aldrich, Merck KGaA, Darmstadt, Germany). One mg powder was dissolved in 0.387 mL of DMSO to prepare a 10 mM stock solution. The prepared stock solutions were stored at −20 °C in small volumes. A new stock solution was used to prepare the working dilutions for each experiment.

### 2.3. Cell Viability Assay

Cell metabolic activity was assessed using the 3-(4,5-dimethylthiazol-2-yl)-5-(3-3-carboxymethoxyphenyl)-2-(4-sulfo-phenyl)-2H-tetrazolium (CellTiter 96^®^ AQueous One Solution Cell Proliferation Assay catalog number G3582, Promega, Milan, Italia). After 48 h of treatment with RSV, 3-MET-OX, and ISOR-H-PG (25–200 µM) in monolayer cultures, MTS was performed with a 4 h incubation at 37° C in a humidified atmosphere containing 5% CO_2_. The absorbance was then measured at 490 nm using a microplate reader (SpectroStar Nano, BMGLABTECH, Ortenberg, Germany). Data were analyzed with Mars Software v3.00R3 (BMG LABTECH, Offenburg, Germany) and plotted as growth curves via GraphPad software (GraphPad 5.0, La Jolla, CA, USA).

### 2.4. Cell Cycle Distribution Analysis

After treatment at sub-cytotoxic doses (IC30, specific for each line), single-cell suspensions of Nthy-ori 3-1, SW1736, and 8505c cells were prepared, and DNA content analysis was performed according to Nicoletti’s protocol. Briefly, 1 × 10^6^ cells were fixed in 70% ethanol, washed with phosphate-buffered saline (PBS), and then resuspended in a DNA extraction buffer containing 0.2 M NaHPO_4_ and 0.1% Tritonx-100 (pH 7.8). After staining with 1 µg/mL propidium iodide for 5 min, fluorescence intensity was determined by FACS Calibur flow cytometer (Becton-Dickinson, East Rutherford, NJ, USA). For each sample, at least 10,000 single-cell events were acquired for cell-cycle analysis. Data acquisition was performed with CellQuest software (v5.x) (Becton-Dickinson, East Rutherford, NJ, USA), and cell cycle distribution (G1, S, and G2/M phase percentages) was calculated using MODFIT-LT 2.0 software program (Verity Software House, Topsham, ME, USA).

### 2.5. Quantitative Real-Time-PCR (qRT-PCR)

Total RNA was extracted from Nthy-ori 3-1, SW1736, and 8505c cell lines via the RNeasy Kit (Qiagen, Hamburg, Germany), according to the manufacturer’s instructions. The qRT-PCR amplification was performed using the QuantiTect SYBR Green PCR Kit (cod. 204243, Qiagen, Hamburg, Germany) on the Rotor-Gene Q instrument (Qiagen, Hamburg, Germany). Specific amplification was verified by analysis of melting curve profiles at the end of each reaction. Relative mRNA expression for each gene was analyzed using the ΔΔCt method according to the guidelines of Livak and Schmittgen and normalized for β-actin expression [15]. The results were plotted as bar graphs with GraphPad Prism 5.0 software (GraphPad Software, La Jolla, CA, USA). The primers used for qRT-PCR reactions are listed in Table 1. All experiments were performed in three independent experiments, each including technical triplicates.

### 2.6. Statistical Analysis and Graphical Representation

Data were expressed as mean ± standard deviation (SD). The appropriate one-way ANOVA with the Bonferroni multiple comparison test was used for statistical comparison between groups. Differences were considered statistically significant at *p* < 0.05 with a 95% confidence interval (* *p* < 0.05, ** *p* < 0.01, *** *p* < 0.001, **** *p* < 0.0001). Analyses and representation as bar graphs (mean ± SD) were performed with the GraphPad Prism 5.0 software (San Diego, CA, USA)

## 3. Results

### 3.1. The Cytostatic Effects of Polyphenol Treatments on ATC Cell Lines

Cell viability was determined by MTS assay after 48 h of treatment with RSV, 3-MET-OX, and ISOR-H-PG (25–200 µM) in monolayer cultures of ATC cell lines (8505c and SW1736) and non-tumor control (Nthy-ori 3-1). According to the results, none of the three polyphenols reduced cell viability in ATC lines. Indeed, the IC_50_ values of RSV, 3-MET-OX, and ISOR-H-PG in ATC cell lines were in the range of ~15–70 µM, with ISOR-H-PG showing the lowest IC_50_ (15.9 µM) and RSV the highest (68.51 µM) in SW1736 cells (Supplementary Figure S1).

**Figure 1:**
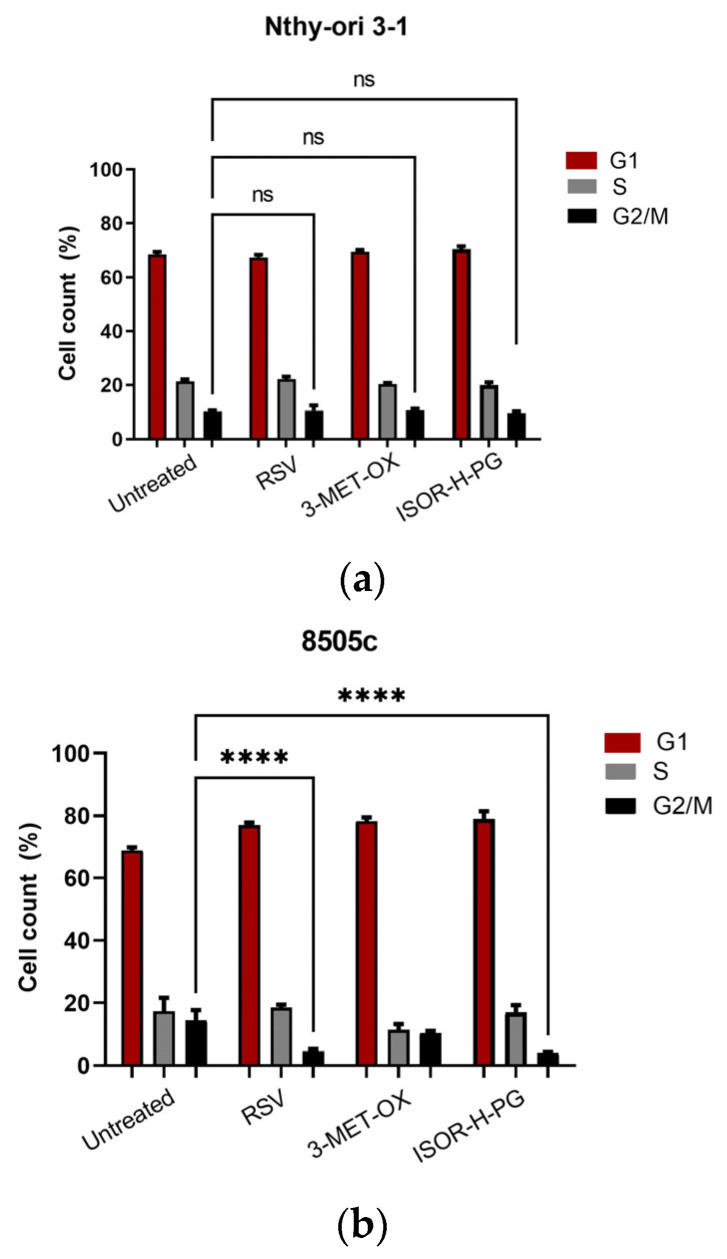

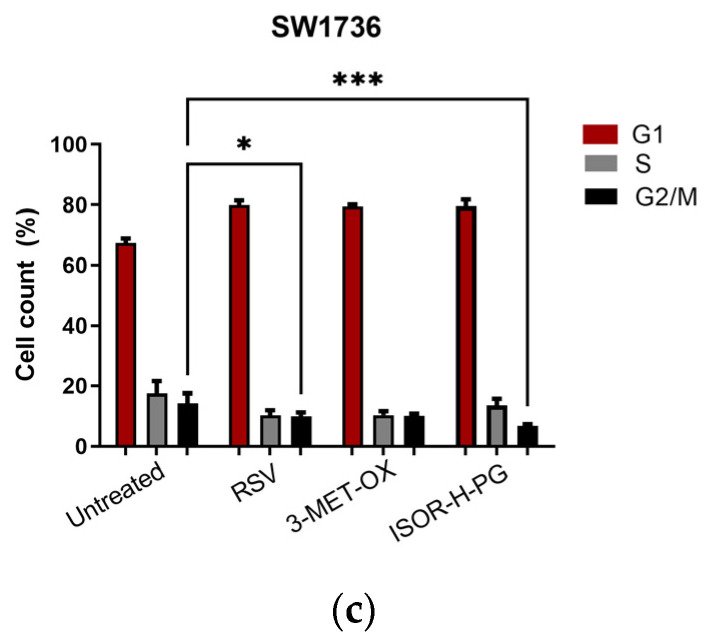
Cell cycle distribution after 48 h exposure to polyphenols at sub-cytotoxic doses (IC_30_) in ATC and non-tumoral control cell lines: representative histograms from flow cytometry analysis showing cell cycle distribution in (**a**) SW1736, (**b**) 8505c (**b**), and (**c**) Nthy-ori 3-1 cells after treatment with RSV, 3-MET-OX, and ISOR-H-PG. Quantitative analysis of G1, S, and G2/M phase percentages, evidencing a significant accumulation of ATC cells in G1 phase and a reduction in S and G2/M phases after polyphenol treatment. Experiments were performed in technical triplicates (n = 3). Data are expressed as the mean ± SD; * *p* = 0.0494; *** *p* = 0.0006; **** *p* < 0.0001; ns = not significant.

These results are consistent with the hypothesis that such compounds primarily act in a cytostatic rather than cytotoxic manner, influencing cell cycle and differentiation programs rather than triggering early apoptosis. The cell cycle distribution pattern was investigated by flow cytometry after 48 h of polyphenol exposure with sub-cytotoxic doses (IC_30_) calculated for each line (Supplementary Figure S1). IC30 was selected to preserve the viability and metabolic activity of treated cells, allowing the assessment of regulatory effects independently from overt cytotoxicity. In both ATC cell lines, all three compounds induced a cell cycle delay, as evidenced by the significantly different cell distribution in the G1, S, and G2/M phases in treated cells compared to each untreated control. In SW1736 cells, the G1 fraction was improved from 67.403% ± 1.237% in untreated controls to 79.833% ± 1.594% with RSV (*p* < 0.0001), 79.393% ± 0.699% with 3-MET-OX (*p* < 0.0001), and 79.510% ± 2.276% with ISOR-H-PG (*p* < 0.0001). Similarly, in 8505c cells, G1 phase percentages increased from 68.890% ± 0.914% in untreated controls to 77.040% ± 0.815 with RSV (*p* = 0.0003), 78.330% ± 1.185% with 3-MET-OX (*p* < 0.0001), and 79.077% ± 2.377% with ISOR-H-PG (*p* < 0.0001). This G1 arrest was paralleled by a reduction in the S phase, which decreased from 17.440% ± 4.220% in untreated SW1736 cells to 10.253% ± 1.796% with RSV (*p* = 0.0009), 10.383% ± 1.351% with 3-MET-OX (*p* = 0.0011), and 13.667% ± 2.240% with ISOR-H-PG (*p* = 0.1095); and from 17.323% ± 1.748% in untreated 8505c cells to 11.350% ± 1.838% with 3-MET-OX (*p* = 0.0085) and, although not significantly, 18.437% ± 0.958% with RSV (*p* > 0.9999) and 16.910% ± 2.372% with ISOR-H-PG (*p* > 0.9999). The G2/M phase was reduced from 14.310% ± 0.984% in untreated SW1736 cells to 9.917% ± 1.370% with RSV (*p* = 0.0494), 10.203% ± 0.701% with 3-MET-OX (*p* = 0.0719), and 6.823% ± 0.582% with ISOR-H-PG (*p* = 0.0006); and in 8505c from 14.333% ± 0.732% to 4.533% ± 0.472% with RSV (*p* < 0.0001), 10.303% ± 0.621% with 3-MET-OX (*p* = 0.1372), and 4.037% ± 0.226% with ISOR-H-PG (*p* < 0.0001). Interestingly, in non-tumoral Nthy-ori 3-1 cells, cycle distribution was not significantly different after polyphenol treatments. The G1 population remained essentially stable (67.3% ± 1.015% with RSV, *p* = 0.3903; 69.4% ± 0.737% with 3-MET-OX, *p* = 0.4446; 70.4% ± 1.069% with ISOR-H-PG, *p* = 0.0449), as did the S phase (22.173% ± 0.925% with RSV, *p* = 0.6033; 20.350% ± 0.477% with 3-MET-OX, *p* = 0.4524; 20.017% ± 1.030% with ISOR-H-PG, *p* = 0.2374) and G2/M (10.527% ± 1.933% with RSV, *p* = 0.9748; 10.680% ± 0.623% with 3-MET-OX, *p* = 0.9122; 9.583% ± 0.683% with ISOR-H-PG, *p* = 0.7230) vs. untreated (G1 68.387% ± 0.995%; S 21.353% ± 0.784%; G2/M 10.263% ± 0.327%) suggesting a specific action of polyphenols for ATC lines (Figure 1).

In summary, treatments with polyphenols caused a slowdown in the cell cycle with accumulation in G1 and reduction in the fraction in S and G2/M, without evidence of marked apoptosis, as suggested by the absence of a pre-G0/G1 peak (Supplementary Figure S2); these results indicate a predominantly cytostatic effect, which prompted further analysis of the impact of the compounds on the state of differentiation and stemness markers.

### 3.2. Basal Expression of Stemness and Thyroid Differentiation Markers in ATC Cell Lines

**Figure 2:**
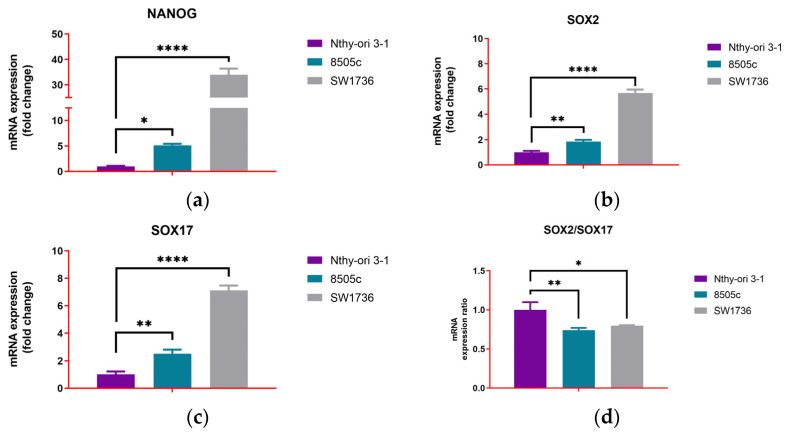
Basal mRNA expression of stemness and differentiation markers in thyroid cell lines. (**a**) NANOG, (**b**) SOX2, (**c**) SOX17, and (**d**) SOX2/SOX17 ratio mRNA expression levels. Experiments were performed in technical triplicates (n = 3). Data are expressed as the mean ± SD and as fold change relative to non-tumoral Nthy-ori 3-1 cells; * *p* < 0.05; ** *p* < 0.01; **** *p* < 0.0001.

To further characterize the biological state of ATC cells, we next evaluated the expression of stemness and thyroid differentiation markers in basal conditions. In both ATC-derived cell lines, a clear stem-like and dedifferentiated profile was observed when compared to the non-tumoral Nthy-ori 3-1 control cell line. NANOG expression was upregulated in SW1736 (34.007 ± 2.406-fold change (fc), *p* < 0.0001) and in 8505c (5.163 ± 0.295-fc, *p* = 0.0327), relative to the low basal levels observed in Nthy-ori 3-1 (1.007 ± 0.110-fc) (Figure 2a). A similar trend was observed for SOX2, which significantly increased in SW1736 (5.690 ± 0.265-fc, *p* < 0.0001) and 8505c (1.857 ± 0.140-fc, *p* = 0.0040) compared to controls (1.003 ± 0.115) (Figure 2b). Unexpectedly, the differentiation-associated marker SOX17 was higher in both SW1736 (7.123 ± 0.352-fc, *p* < 0.0001) and 8505c (2.513 ± 0.290, *p* = 0.0023) compared to non-tumoral Nthy-ori 3-1 (1.017 ± 0.215) (Figure 2c). However, the SOX2/SOX17 ratio was lower in ATC cell lines (SW1736: 0.798 ± 0.007-fc, *p* = 0.0116; 8505c: 0.741 ± 0.029-fc, *p* = 0.0034) compared to the Nthy-ori 3-1 cell line (1.000 ± 0.097), indicating a stem-like, de-differentiated program despite higher SOX17 levels (Figure 2d).

**Figure 3:**
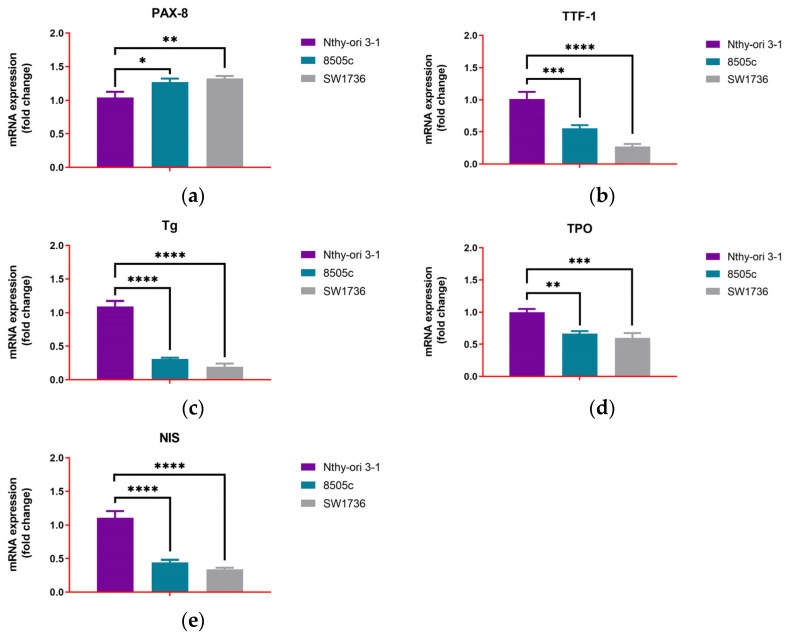
Basal mRNA expression of thyroid differentiation markers in thyroid cell lines. (**a**) PAX-8, (**b**) TTF-1, (**c**) Tg, (**d**) TPO, and (**e**) NIS mRNA expression levels. Experiments were performed in technical triplicates (n = 3). Data are expressed as the mean ± SD and as fold change relative to non-tumoral Nthy-ori 3-1 cells; * *p* < 0.05; ** *p* < 0.01; *** *p* < 0.001; **** *p* < 0.0001.

ATC cell line expression profiles were compared to those of non-tumoral Nthy-ori 3-1 cells to assess their dedifferentiated phenotype. Concerning thyroid-specific transcription factors and functional genes, SW1736 cells showed a drastic reduction of TTF-1 (0.273 ± 0.040-fc, *p* < 0.0001), almost complete loss of Tg (1.197 ± 0.045-fc, *p* < 0.0001), TPO (0.597 ± 0.078-fc, *p* = 0.0005), and NIS (0.340 ± 0.026-fc, *p* < 0.0001), but displayed relatively preserved or even increased expression of PAX-8 (1.323 ± 0.038-fc, *p* = 0.0042). 8505c cells also exhibited a strong downregulation of Tg (0.310 ± 0.020-fc, *p* < 0.0001), TPO (0.663 ± 0.042-fc, *p* = 0.0012), and NIS (0.447 ± 0.035-fc, *p* < 0.0001), but retained a detectable expression of TTF-1 (0.557 ± 0.047-fc, *p* = 0.0008) and lower levels of PAX-8 (1.270 ± 0.053-fc, *p* = 0.0119) compared to SW1736 (Figure 3).

### 3.3. The Loss of Spheroid Morphology in ATC 3D Cell Cultures

**Figure 4:**
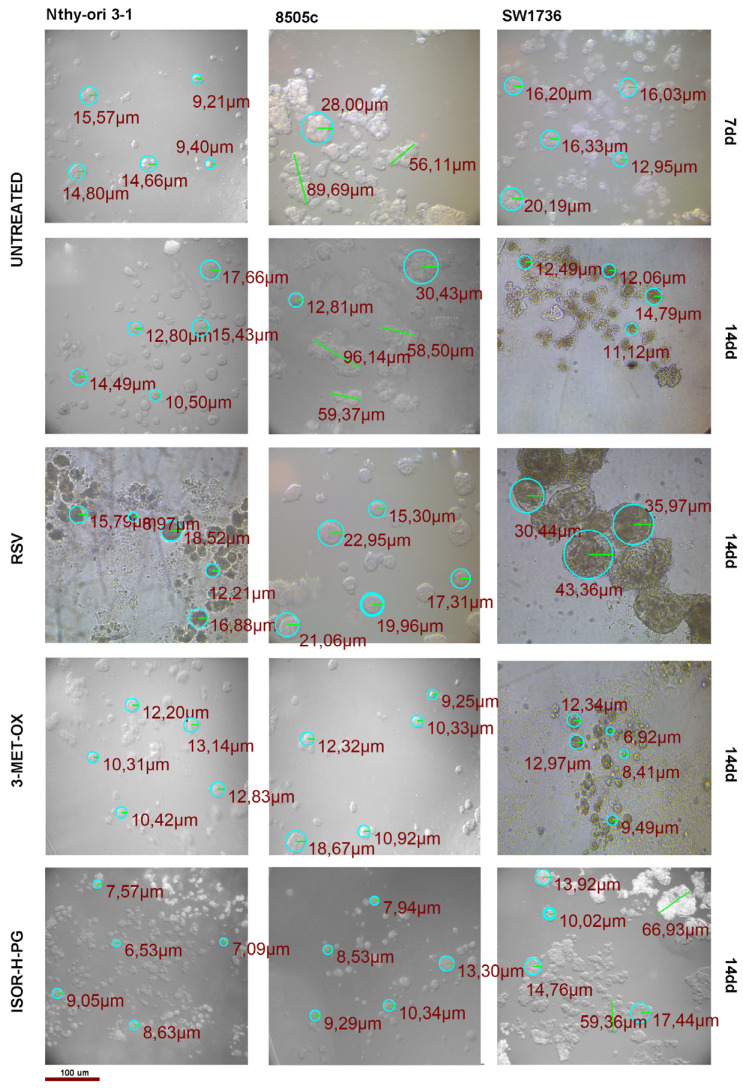
Representative images of 3D spheroid cultures of ATCs, 8505c and SW1736, and Nthy-ori 3-1 cell lines after 14 days of treatment with RSV, 3-MET-OX, and ISOR-H-PG, showing morphological and dimensional characterization of spheroid in thyroid cell lines compared to control (untreated). Experiments were performed in technical triplicates (n = 3). Scale bar 100 µm.

To better mimic the tumor microenvironment in vivo, ATC cell lines (8505c and SW1736) and non-tumor Nthy-ori 3-1 cells were cultured in 3D spheroid systems and treated with RSV, 3-MET-OX, and ISOR-H-PG. Under basal conditions, non-tumoral Nthy-ori 3-1 cells formed compact and well-organized spheroids, consistent with their differentiated epithelial phenotype and the maintenance of tight cell–cell junctions and structural polarity; whereas ATC-derived spheroids exhibited a looser and irregular organization, reflecting the loss of adhesion properties and tissue architecture typical of highly dedifferentiated tumors. After 14 days of treatment, the ATC cell spheroids gradually acquired a more spherical and homogeneous morphology, suggesting a restoration of epithelial-like features (Figure 4). These morphological alterations were most pronounced after RSV and 3-MET-OX treatment, whereas ISOR-H-PG exerted milder effects. In SW1736 spheroids, RSV treatment resulted in a slight increase in mean radius; however, despite the modest enlargement, the spheroid appeared more regular and compact in comparison to the untreated condition. In contrast, in 8505c spheroids all three polyphenol treatments induced a reduction in mean spheroid size, reflecting a consistent decrease in radial dimensions (Supplementary Figure S3).

Notably, non-tumoral Nthy-ori 3-1 spheroids preserved their architecture under all conditions, revealing a potential selective action of the compounds on malignant cells.

### 3.4. Evaluation of Stemness Marker Expression After Polyphenol Treatments in ATC 3D Spheroid Culture

**Figure 5:**
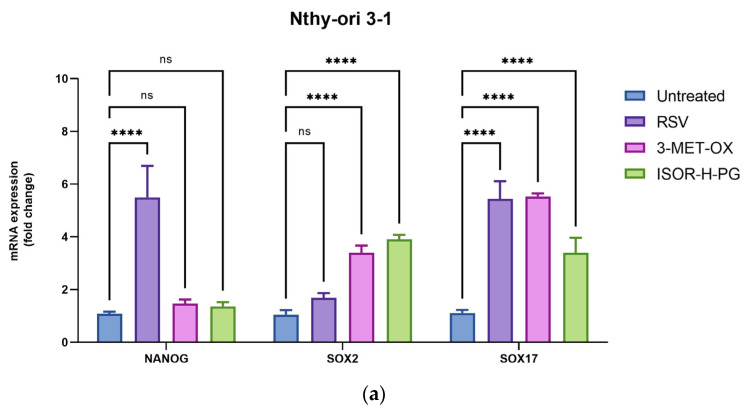

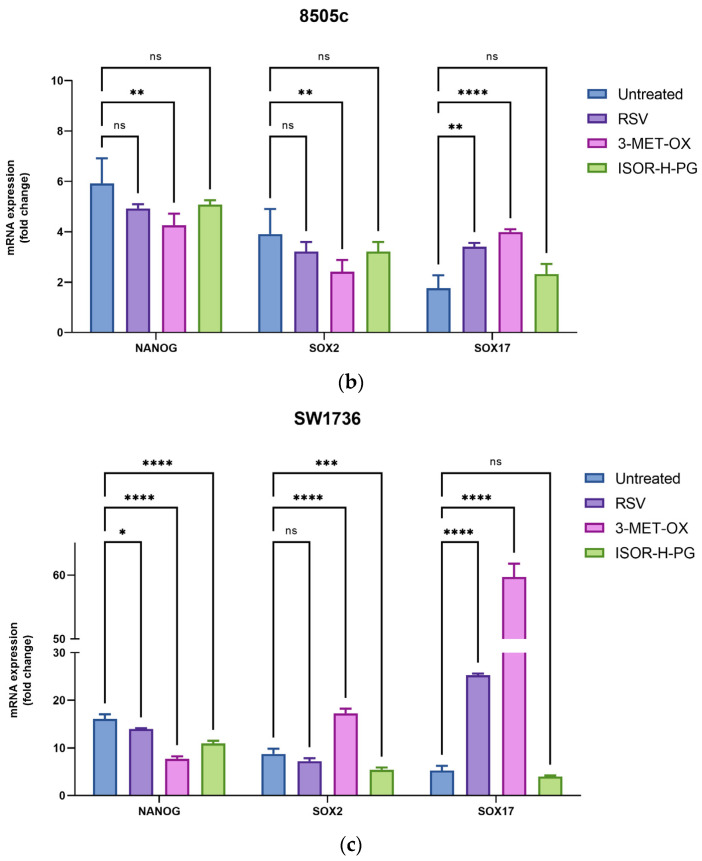
mRNA expression of stemness and differentiation markers in 3D spheroid cultures of thyroid cell lines after treatment with RSV, 3-MET-OX, and ISOR-H-PG. Each graph reports NANOG, SOX2, and SOX17 gene expression levels in (**a**) Nthy-ori 3-1, (**b**) 8505c, and (**c**) SW1736 cell lines. Experiments were performed in technical triplicates (n = 3). Data are expressed as the mean ± standard deviation and as fold change relative to Nthy-ori untreated controls; * *p* < 0.05; ** *p* < 0.01; *** *p* < 0.001; **** *p* < 0.0001; ns = not significant.

To assess whether polyphenol treatments could influence the stem cell properties of ATC cells in a 3D context, the gene expression of stemness markers (NANOG and SOX2) and differentiation markers (SOX17) was assessed after treatment with RSV, 3-MET-OX, and ISOR-H-PG in spheroids of ATC cell lines and non-tumor controls (Figure 5). In 8505c cells, NANOG expression also decreased after exposure to all polyphenols; however, statistical significance was reached only with 3-MET-OX (5.920 ± 1.000-fc untreated vs. 4.260 ± 0.464-fc, *p* = 0.002), whereas the reductions induced by RSV (4.927 ± 0.168-fc, *p* = 0.0846) and ISOR-H-PG (5.080 ± 0.173-fc, *p* = 0.1797) did not achieve significance (Figure 5b). In SW1736 cells, NANOG expression was significantly reduced following all three treatments compared to untreated controls (16.067 ± 1.002-fc), with the strongest downregulation observed after 3-MET-OX (7.757 ± 0.511-fc, *p* < 0.0001) and ISOR-H-PG (10.910 ± 0.572-fc, *p* < 0.0001). At the same time, RSV exerted a minor but still significant effect (13.933 ± 0.179-fc, *p* = 0.0260) (Figure 5c).

In line with these findings, the analysis of SOX2 revealed a differential response to polyphenol treatments. In 8505c spheroids, SOX2 expression (3.910 ± 1.000 untreated) was significantly reduced only by 3-MET-OX (2.415 ± 0.466, *p* = 0.0053), whereas the reductions induced by RSV (3.213 ± 0.386-fc, *p* = 0.3435) and ISOR-H-PG (3.213 ± 0.386-fc, *p* = 0.3435) were not significant (Figure 5b). In SW1736 spheroids, SOX2 expression (8.770 ± 1.110-fc in untreated controls) was modestly reduced by RSV (7.233 ± 0.655-fc, *p* = 0.1516), significantly downregulated by ISOR-H-PG (5.450 ± 0.464-fc, *p* = 0.0005). However, SOX2 was up-regulated after 3-MET-OX treatment (17.230 ± 1.000-fc, *p* < 0.0001) (Figure 5c).

Regarding SOX17, a differentiation-associated marker, polyphenol treatments promoted its expression in both ATC cell lines. In 8505c spheroids, SOX17 expression (1.757 ± 0.513 untreated) was significantly enhanced by RSV (3.410 ± 0.148-fc, *p* = 0.0021) and 3-MET-OX (3.993 ± 0.111-fc, *p* < 0.0001), but not by ISOR-H-PG (2.313 ± 0.414-fc, *p* = 0.6091) (Figure 5b). In SW1736 spheroids, basal SOX17 levels (5.280 ± 1.000 untreated) increased after RSV (25.220 ± 0.390-fc, *p* < 0.0001) and more strongly after 3-MET-OX (59.707 ± 2.082-fc, *p* < 0.0001), while ISOR-H-PG did not induce a significant effect (4.003 ± 0.214-fc, *p* = 0.3004) (Figure 5c).

Together, these data indicate that while all three polyphenols modulate the stemness and differentiation profile of ATC spheroids, RSV and 3-MET-OX exert the most consistent and pronounced effects.

### 3.5. Impact of Polyphenols on the SOX2/SOX17 Balance in ATC Spheroids

**Figure 6:**
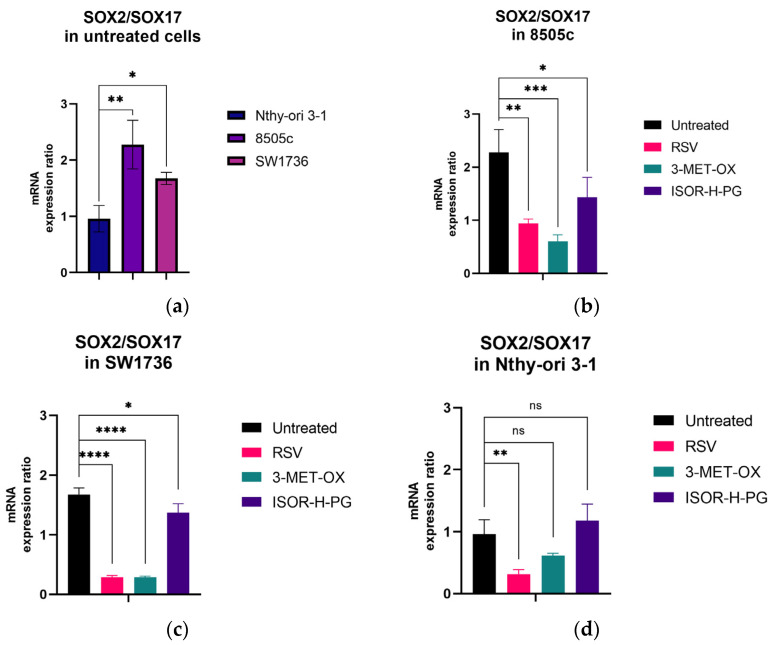
SOX2/SOX17 expression ratio in 3D spheroid cultures of thyroid cell lines after treatment with RSV, 3-MET-OX, and ISOR-H-PG. (**a**) Basal SOX2/SOX17 expression ratio in untreated thyroid cell lines. Polyphenol effect treatment on SOX2/SOX17 expression ratio in (**b**) 8505c and (**c**) SW1736 cells (**d**) Nthy-ori 3-1 spheroids. The SOX2/SOX17 expression ratio was calculated as the ratio between the relative fold change in each gene compared to untreated Nthy-ori. Experiments were performed in technical triplicates (n = 3). Data are expressed as the mean ± standard deviation; * *p* < 0.05; ** *p* < 0.001; *** *p* = 0.0004; **** *p* < 0.0001, ns = not significant.

In 3D cultures of both ATC lines, the SOX2/SOX17 expression ratio is significantly higher than in non-cancerous Nthy-ori 3-1 cells (SW1736: 1.675 ± 0.109, *p* = 0.0463; 8505c: 2.276 ± 0.432, *p* = 0.0043 vs. Nthy-ori 3-1), confirming a stem and dedifferentiated phenotype (Figure 6a).

After treatment with the three polyphenols, a reduction in the SOX2/SOX17 expression ratio was observed, with specific differences depending on the molecule used (Figure 6b,c). Specifically, RSV induced a significant reduction in the SOX2/SOX17 expression ratio in ATC spheroids compared to untreated control (SW1736: 0.287 ± 0.030 vs. 1.675 ± 0.109, *p* < 0.0001; 8505c: 0.941 ± 0.083 vs. 2.276 ± 0.432, *p* < 0.0001). Likewise, after treatment with 3-MET-OX, the SOX2/SOX17 expression ratio was decreased in the 8505c (0.605 ± 0.121 vs. 2.276 ± 0.432 untreated, *p* < 0.0001) and SW1736 (0.289 ± 0.017 vs. 1.675 ± 0.109 untreated, *p* < 0.0001) spheroids. ISOR-H-PG also induced a decrease in the SOX2/SOX17 expression ratio in 8505c spheroids (1.431 ± 0.376 vs. 2.276 ± 0.432, *p* < 0.0001), and in SW1736 (1.365 ± 0.155 vs. 1.675 ± 0.109, *p* = 0.0131) (Figure 6b,c). Interestingly, in 3D cultures of Nthy-ori 3-1 control cells the effect of polyphenols on the SOX2/SOX17 ratio is lower (RSV: 0.316 ± 0.070, *p* = 0.0026) or not significant (3-MET-OX: 0.316 ± 0.038, *p* = 0.1298; ISOR-H-PG: 1.179 ± 0.266, *p* = 0.4362) compared to untreated cells (0.958 ± 0.235) (Figure 6d).

### 3.6. Expression of Thyroid Differentiation Markers After Treatment with Polyphenols in ATC Compared to Non-Tumoral Control

To evaluate the effect of polyphenols on the ability to induce a more differentiated phenotype in ATC cells, the expression of the main thyroid markers TPO, TTF-1, and NIS was analyzed after treatment with polyphenols in 3D cultures. Expression levels were normalized relative to the untreated control of each cell line, allowing for direct comparison of treatment effects between tumor and non-tumor spheroids. Considering the previous results, attention was focused primarily on RSV and 3-MET-OX.

**Figure 7:**
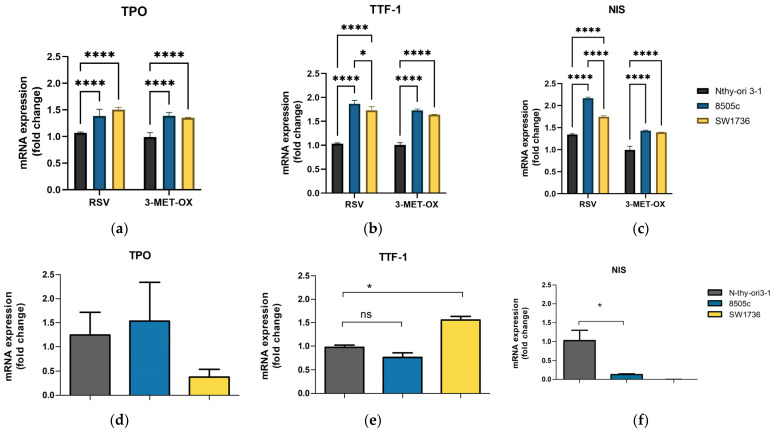
Polyphenol treatments promote thyroid differentiation in 3D spheroid cultures. The bar graphs show mRNA expression levels of (**a**) TPO; (**b**) TTF-1; and (**c**) NIS in Nthy-ori 3-1, 8505c, and SW1736 spheroids after treatment with RSV and 3-MET-OX; (**d**) TPO; (**e**) TTF-1; and (**f**) NIS in Nthy-ori 3-1, 8505c, and SW1736 spheroids at basal levels. Experiments were performed in technical triplicates (n = 3). Data are expressed as the mean and standard deviation of three independent experiments; * *p* < 0.05; **** *p* < 0.0001; ns = not significant.

In both ATC lines, SW1736 and 8505c, the treatments resulted in significantly higher expression of TPO compared to non-tumor Nthy-ori 3-1 control cells. In 8505c spheroids, the TPO gene is expressed 29.5% after RSV (1.383 ± 0.130-fc, *p* < 0.0001) and 40.7% after 3-MET-OX treatment (1.504 ± 0.0421.383 ± 0.130-fc, *p* < 0.0001) more highly than in control cells, while in SW1736 the increase was + 40.9% with RSV (1.504 ± 0.042-fc, *p* < 0.0001) and +37.0% with 3-MET-OX (1.352 ± 0.008-fc, *p* < 0.0001) (Figure 7a). After RSV treatment, TTF-1 gene expression increased by 68.8% (1.727 ± 0.078-fc, *p* < 0.0001) in SW1736 and 81.8% (1.863 ± 0.075-fc, *p* < 0.0001) in 8505c compared to the control. The 3-MET-OX treatment increased TTF-1 expression by 60.8% (1.638 ± 0.006-fc, *p* < 0.0001) in SW1736 and 70.3% (1.725 ± 0.035-fc, *p* < 0.0001) in 8505c compared to Nthy-ori 3-1 (Figure 7b). NIS expression levels increased by 16.6% in SW1736 cells (1.745 ± 0.027-fc, *p* < 0.0001) and by 61.4% in 8505c cells (2.170 ± 0.020-fc, *p* < 0.0001) compared with the control following RSV treatment. After 3-MET-OX treatment, NIS expression was up-regulated by 36.9% in SW1736 cells (1.394 ± 0.030-fc, *p* < 0.0001) and by 40.7% in 8505c cells (1.428 ± 0.018-fc, *p* < 0.0001) compared to the control (Figure 7c). Interestingly, before treatment, thyroid differentiation markers displayed an expression pattern fully consistent with the de-differentiated phenotype of ATC spheroids compared to Nthy-ori 3-1 cells. While TPO did not show significant modulation, both TTF-1 and NIS were markedly altered. In 8505c spheroids, TTF-1 expression was strongly reduced (0.1293 ± 0.02003-fc, *p* < 0.05) with no detectable expression in SW1736, whereas NIS expression was significantly downregulated in 8505c (0.7633 ± 0.09504-fc, *p* < 0.05) and conversely upregulated in SW1736 (1.557 ± 0.07767-fc, *p* < 0.05) compared to Nthy-ori 3-1 spheroids.

## 4. Discussion

Phytochemicals have been increasingly evaluated for different purposes, especially in the health field. They have been tested to improve therapeutic responses or offer alternative treatment for acute or chronic diseases, including cancer, or to prevent them. Indeed, many lines of investigation are currently ongoing, but only a limited number of natural compounds have already been made available in clinical practice for treating diseases or metabolic dysfunctions [16,17,18].

Specifically, resveratrol (RSV) and its derivatives–such as 3,4′,5-trimethoxystilbene (3-MET-OX) and isorhapontigenin (ISOR-H-PG)-were widely explored for their anti-aging, anti-inflammatory, and anti-diabetic effects, and for their protective effects towards renal, liver, or cardiovascular functions. Moreover, some in vitro and in vivo studies have successfully highlighted their possible anti-tumor effect, making them relevant for drug development and for the discovery of new therapeutic strategies. These compounds can exert their effect both by affecting the migration and proliferation of tumoral cell-promoting apoptosis and acting at a molecular level (e.g., influencing epigenetic mechanisms) [19,20,21,22,23].

This work aimed to evaluate how RSV and RSV-derived nutraceuticals could limit the proliferation of CSCs and then their ability to allow aggressive tumors, such as ATC, to grow and spread via metastatic processes. Indeed, we decided to focus also on 3D cultures of ATC to reproduce a tumoral model more reliable than the traditional 2D cultures, especially for testing the effects of drugs and compounds. Nowadays, several methods to establish thyroid cancer in 3D cultures have been proposed, including spheroids. While the complexity of spheroids is quite limited compared to other 3D cultures (such as organoids), they are optimal to test therapeutics in preclinical settings because they can mimic tumor cell interactions and the oxygen gradients typical of tumoral masses [24,25].

As previously stated, ATC is an undifferentiated tumor with a poor prognosis. Thus, we aimed to perform a preliminary evaluation of compounds that could exert a synergistic effect with traditional therapies: specifically, we wanted to prove the ability of resveratrol and its derivatives to induce a regression of the aggressive phenotype a differentiation process, to resensitize CSC to therapeutics, and limit cancer proliferation.

In our study, polyphenol treatments did not induce cell death in monolayer cultures, but they made the ATC cell cultures cycle more slowly compared to the control samples and untreated cells, suggesting a selective effect of polyphenols on the proliferation rate of ATC cells and their undifferentiated phenotype. This observation was confirmed with the 3D culture models of ATC. Specifically, non-tumoral thyroid cells Nthy-ori 3-1 cells formed small and well-organized spheroids, consistent with their differentiated epithelial phenotype. In contrast, ATC-derived spheroids show irregular aggregate formation, consistent with more mesenchymal features. Interestingly, following treatment with resveratrol and its analogs, ATC spheroids gradually acquired a more spherical and homogeneous morphology, reflecting a restoration of epithelial-like features.

We sought to correlate the changes in ATC cells to a possible shift from an undifferentiated to a more differentiated gene expression profile, with a reduction in self-renewal ability. This hypothesis came after some observations:

- Nthy-ori 3-1 cells-mature and differentiated cells obtained from the immortalization of normal human follicular epithelial cells and often used as a control compared to ATC cells [26]-did not change their growth rate after polyphenol treatments.
- The growth and invasiveness of ATC could be related to the high proliferation rate of its drug-resistant CSCs [27]. Thus, a reduction in proliferation could be caused by the improvement of differentiation and the reduction in CSC stemness.
- As previously stated, in vitro studies showed that RSV can promote the differentiation towards the epithelial lineage by modulation of the SOX2/SOX17 balance [13].

Accordingly, we decided to focus on the expression of thyroid functional genes related to differentiated follicular cells, such as NIS, Tg, and TPO, and on genes coding for transcription factors involved in the activation of such genes, such as TTF-1 and PAX-8 [28,29]. Moreover, we also considered genes related to stemness, such as NANOG and SOX2, whose regulation is crucial for self-renewal and cell commitment [6,30,31]. Specifically, the latter was evaluated because of its well-known role in different kinds of cancers, such as breast and lung, and thyroid tumors, even if the exact mechanisms are still under investigation [32,33,34,35,36,37].

SOX2 is part of a group of genes whose transcription plays a relevant role in human organogenesis and development; most of them also have a role in tumorigenesis and metastasis. Altogether, they compose the so-called SOX family, including 20 genes of coding for transcription factors with DNA-binding domains. SOX genes are often deregulated in tumors, and every member of the family has a specific mechanism of action. Some of them, such as SOX17, are tumor suppressor genes. SOX17 exerts its action via the inhibition of the Wnt/β-catenin signaling pathway in cancer, and its methylation is related to the development of breast and colorectal cancers, and to PTC progression. Moreover, it is a regulator of endothelial cell differentiation, which implies that it could be involved in the tumoral angiogenesis process. For its role as a tumor suppressor and for its involvement in epithelial–mesenchymal transition (EMT)—in which the relationship with SOX2 can influence the proliferation and progression of different cancers—we decided to focus on SOX2/SOX17 balance to verify if it can determine a differentiation process in ATC cells [38,39,40,41,42].

The biological and functional significance of the SOX2/SOX17 balance is supported by Stefanovic et al. that describe a cooperation at physiological levels between SOX2 and Oct4 in sustaining the Oct4/SOX2/Nanog transcriptional loop and preserving pluripotency [43]. Conversely, when SOX2 levels decline, Oct4 preferentially engages the SOX17 regulatory region, inducing SOX17 transcription and redirecting cells toward lineage specification. Aksoy et al. showed that retinoic acid promotes this transcriptional switch, driving cells toward endo- and mesodermal fates [44]. Consistently, Seguin et al. demonstrated the overexpression of SOX17 human embryoid stem cells drives the acquisition of an epithelial morphology while maintaining detectable Nanog levels, thus representing a transitional, differentiation-oriented state rather than full pluripotency [45]. Considering this evidence, the SOX2/SOX17 ratio functions as a key indicator of whether the transcriptional program favors a SOX2-driven stem state or a SOX17-driven commitment state.

Thus, we performed RT-qPCR analyses on monolayer cultures before and after treatments. As expected, before the treatments, the expression of genes involved in thyroid differentiation, such as TTF-1, TPO, and NIS, is significantly reduced both in SW1736 and 8505c cells compared to control ones (Nthy-ori 3-1). The only exception was the expression of PAX-8, which is increased in SW1736 cells: this gene codes for a relevant thyroid transcription factor, and its activity is related to that of TTF-1. We can infer an impaired synergism between the two genes in SW1736 cells, given the more aggressive and undifferentiated phenotype of this cell line compared to 8505c [46,47,48]. Regarding the expression of SOX2 and SOX17, they were overexpressed in both ATC cell lines compared to the control ones, along with NANOG, which is typically overexpressed in stem cells and CSCs, with higher fold-change values observed in SW1736 cells. Indeed, the SOX2/SOX17 expression ratio was lower for ATC cell lines, implying a higher proliferative potential due to stemness features. The same trend was observed in 3D models prior to the administration of treatments.

The treatments with nutraceuticals lead to a different expression of the evaluated genes, suggesting a more differentiated status of ATC cells. Both in 2D and 3D cultures, the SOX2/SOX17 expression ratio was significantly reduced compared to the untreated cells, with the most evident difference after RSV and 3-MET-OX treatment in both 8505c and SW1736 cells. The reduction due to ISOR-H-PG was less effective in SW1736 cells; however, it was statistically significant. This data was also supported by an increase in the expression of TTF-1, NIS, and TPO compared to controls in 3D models after the administration of RSV and its derivatives. The changes induced in gene expression by the phytochemicals resulted in the induction of a more differentiated status in ATC cell lines, with a reduction in their proliferation rate. No such effect was observed for Nthy-ori 3-1 cells, except for a reduction in the SOX2/SOX17 expression ratio and increased expression of NANOG after RSV treatment, although this did not lead to any differences in their cell cycle, proliferation rate, or phenotype.

**Figure 8:**
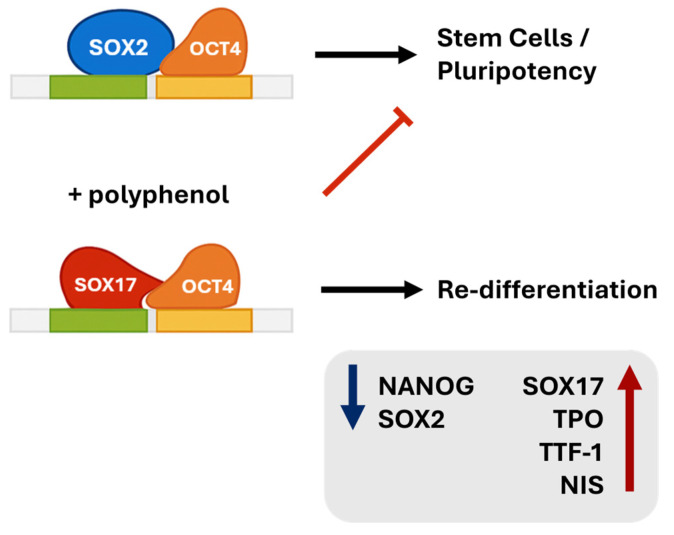
Schematic representation of the effect of RSV on the SOX2/SOX17 balance. The equilibrium between SOX2/OCT4 and SOX17/OCT4 complexes regulates the balance between stemness and differentiation in thyroid cells.

In undifferentiated cells, Octamer-binding Transcription Factor 4 (OCT4) cooperates with SOX2 to support self-renewal via NANOG expression and inhibit maturation, while interaction with SOX17 diverts its transcriptional activity towards cell specification programs. The reduction in the SOX2/SOX17 ratio observed after treatment with RSV or 3-MET-OX suggests that these compounds may favor the transition from SOX2/OCT4 complexes to SOX17/OCT4 complexes, orienting cells towards a more differentiated profile (Figure 8). In this context, polyphenols could act as a modulator of the balance between stemness and differentiation, consistent with the observed increase in thyroid-specific gene (TTF-1, NIS, TPO) expression.

While these results are encouraging, this study has some important limitations. First, data validation via Western blot would be a relevant improvement. However, a Geltrex-free method is necessary—and it is under development—for more precise protein quantification and detection. Moreover, this new approach could allow us to obtain accurate viability data on spheroids (e.g., via MTT or MTS) to confirm our results.

In the future, deepening the modulation of ATC differentiation genes due to nutraceuticals should be another goal of our research, considering analyses such as transcriptomics or proteomics. Currently, even if the effects of resveratrol or other natural compounds are associated with the modulation of cell proliferation and the induction of apoptosis, only an extensive omics-based characterization could explain the exact involvement of the differentiation genes after the treatment of ATC with these compounds. Moreover, this validation needs to be applied both to the 2D and 3D models, to highlight eventual differences in gene modulation and protein expression also in complex microenvironments such as the 3D one [49,50].

## 5. Conclusions

This work highlights the potential role of phytochemicals in future therapies for aggressive thyroid cancers, such as ATC. Their effects could be synergic with traditional therapies, improving their success rate by resensitizing tumoral cells to treatments and overcoming the drug resistance due to CSCs, considered one of the possible mechanisms allowing ATC to cause high mortality. However, this study requires further investigation. First, additional analyses need to confirm whether the observed change in gene expression profile translates into a related change at a protein level. In particular, it is fundamental to explore the modulation of the thyroid-stimulating hormone (TSH) pathway, including the functional activity evaluation of the TSH receptor (its expression and responsiveness). Moreover, to consolidate the translational significance of these findings, ex vivo studies employing thyroid tumor organoids and in vivo experiments using murine models of anaplastic thyroid carcinoma are warranted. Providing a deeper understanding of the mechanisms of action of RSV and its derivatives could enhance their therapeutic implications in ATC.

## Figures and Tables

**Table 1:** Primer sequences.

Genes	Sequence (5′-3′)/Code	Company
NANOG	QT01025850	Qiagen
SOX2	QT00237601	Qiagen
SOX17	QT00204099	Qiagen
PAX-8	QT01010583	Qiagen
TTF-1	QT00010682	Qiagen
Tg	QT00095053	Qiagen
TPO	QT00072982	Qiagen
NIS	F: CTATGGCCTCAAGTTCCTCT	MWG
	R: TCGTGGCTACAATGTACTGC	
β-actin	QT00095431	Qiagen

List of primers used for qRT-PCR analysis. For primers from QuantiTect Primer Assays (Qiagen, Hamburg, Germany), the corresponding GeneGlobe IDs are reported.

## Data Availability

The data that support the findings of this study are available from the corresponding author upon reasonable request.

## Supplementary Materials

The following supporting information can be downloaded at: https://www.mdpi.com/article/10.3390/biology14121730/s1, Figure S1: Cell viability of anaplastic thyroid cancer (ATC) cell lines after treatment with polyphenols. Cell viability was evaluated by MTS assay after 48 h exposure to resveratrol (RSV), 3,4′,5-trimethoxystilbene (3-MET-OX), and isorhapontigenin (ISOR-H-PG) (25–200 µM) in anaplastic thyroid carcinoma cells (8505c and SW1736) and in the nontumoral cell line (Nthy-ori 3-1). Dose–response curves were used to calculate IC50 values by nonlinear regression. Data are presented as mean ± SD of three independent experiments. Figure S2: Representative histograms from flow cytometry analysis showing cell cycle distribution in untreated (CTRL), anaplastic thyroid carcinoma cells (8505c and SW1736), and non-tumoral control cells (Nthy-ori 3-1), after 48 h exposure to a sub-cytotoxic dose (IC_30_) of resveratrol (RSV), 3,4′,5-trimethoxystilbene (3-MET-OX), and isorhapontigenin (ISOR-HPG). Figure S3: Comparative analysis of the size of spheroids (mean radius) of ATC cells (8505c and SW1736) and non-tumour cells (Nthy-ori 3-1) 14 days after treatment with polyphenols (RSV, 3-MET-OX, ISOR-H-PG). Morphometric analyses were performed using NIS-Elements BR software (version 6.20.00, Nikon). The values are derived from the measurement of 5 spheroids per condition. Data are expressed as mean ± SD; ns, not significant; **** *p* < 0.000; ns = not significant.

## Abbreviations

The following abbreviations are used in this manuscript:

3-MET-OX	3,4′,5-Trimethoxy-*trans*-stilbene
ATC	Anaplastic Thyroid Cancer
CSCs	Cancer Stem Cells
EMT	Epithelial–Mesenchymal Transition
ISOR-H-PG	Isorhapontigenin
NANOG	Homeobox Protein NANOG
NIS	Sodium–Iodide Symporter
OCT4	Octamer-binding Transcription Factor 4 (POU5F1)
PAX-8	Paired Box Gene 8
PTC	Papillary Thyroid Cancer
RSV	Resveratrol
SOX17	SRY-related HMG-box 17
SOX2	SRY-related HMG-box 2
Tg	Thyroglobulin
TPO	Thyroid Peroxidase
TTF-1	Thyroid Transcription Factor-1 (NKX2-1)
