# Supplementary material for: SOX2/SOX17 Molecular Switching by Polyphenols to Promote Thyroid Differentiation in 2D and 3D Models of Anaplastic Thyroid Cancer
Source: Biology (Basel). 2025 Dec 2;14(12):1730. doi: 10.3390/biology14121730 (PMC12730977; doi:10.3390/biology14121730)
Supplement: Supplementary file 1 [file biology-14-01730-s001.zip › biology-3968054-supplementary.pdf]

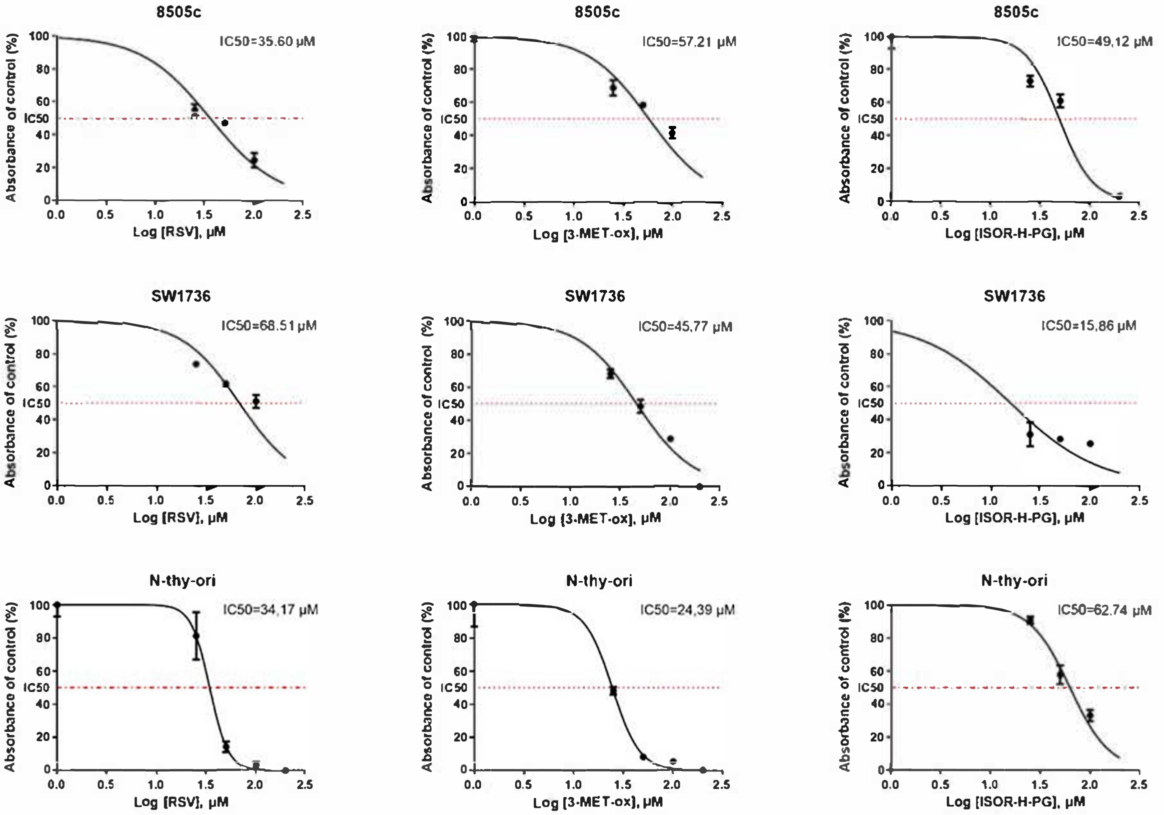

Figure S1. Cell viability of anaplastic thyroid cancer (ATC) cell lines after treatment with polyphenols. Cell viability was evaluated by MTS assay after 48 h exposure to resveratrol (RSV), 3,4′,5-trimethoxystilbene (3-MET-OX), and isorhapontigenin (ISOR-H-PG) (25–200 μM) in anaplastic thyroid carcinoma cells (8505c and SW1736) and in the non-tumoral cell line (Nthy-ori 3-1). Dose–response curves were used to calculate IC_{50} values by nonlinear regression. Data are presented as mean ± SD of three independent experiments.

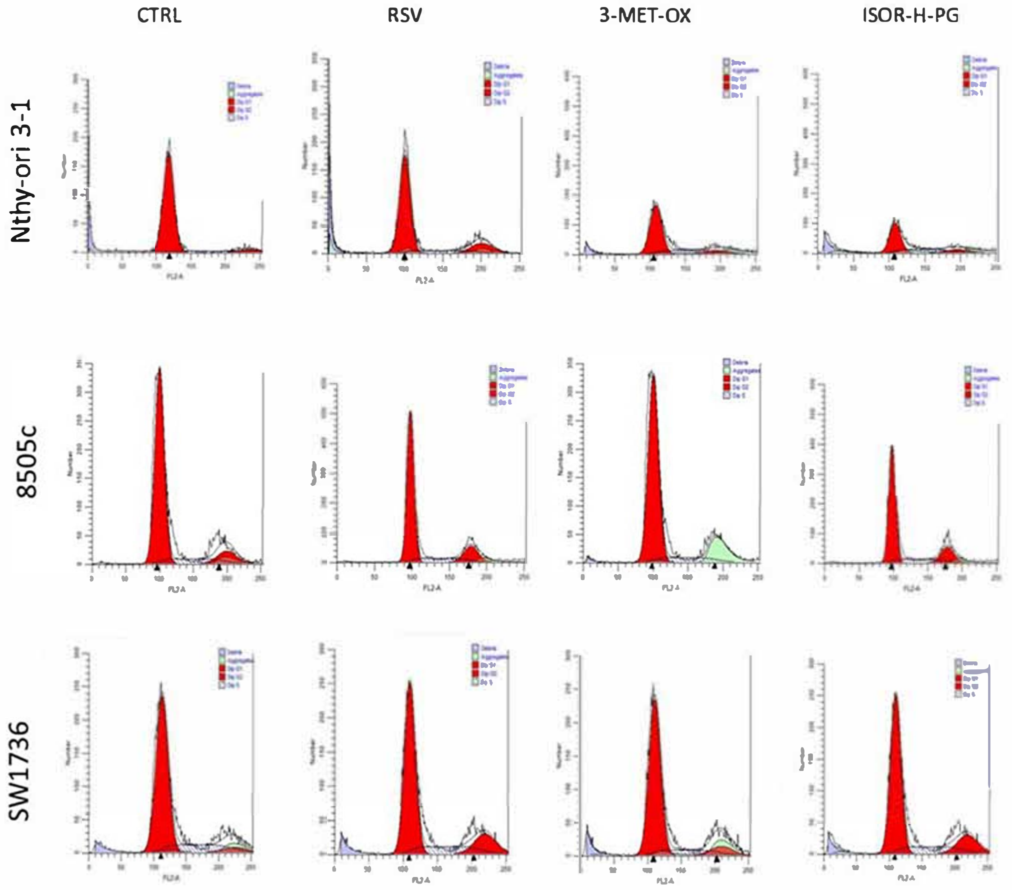

Figure S2. Representative histograms from flow cytometry analysis showing cell cycle distribution in untreated (CTRL), anaplastic thyroid carcinoma cells (8505c and SW1736), and non-tumoral control cells (Nthy-ori 3-1), after 48 h exposure to a sub-cytotoxic dose (IC_{30}) of resveratrol (RSV), 3,4′,5-trimethoxystilbene (3-MET-OX), and isorhapontigenin (ISOR-H-PG).

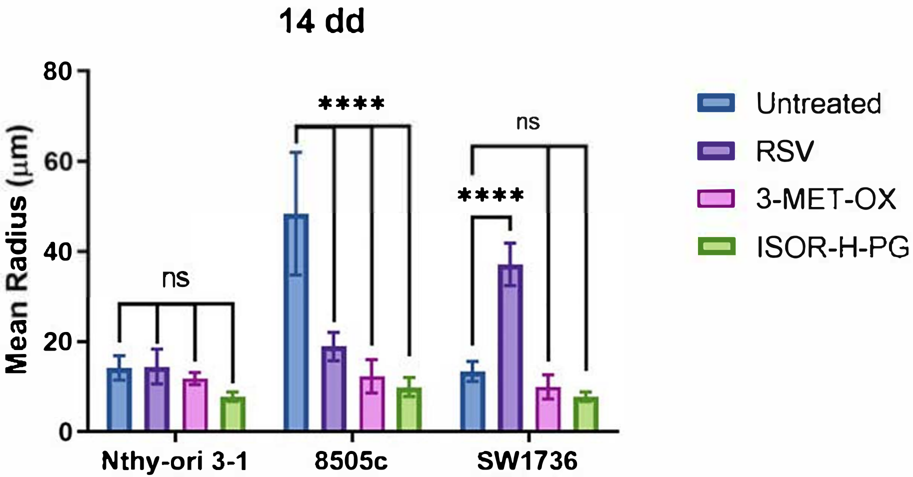

Figura S3. Comparative analysis of the size of spheroids (mean radius) of ATC cells (8505c and SW1736) and non-tumour cells (Nthy-ori 3-1) 14 days after treatment with polyphenols (RSV, 3-MET-OX, ISOR-H-PG). Morphometric analyses were performed using NIS-Elements BR software (version 6.20.00, Nikon). The values are derived from the measurement of 5 spheroids per condition. Data are expressed as mean ± SD; ns, not significant; **** $p < 0.0001$
